# Pre-Birth and Early-Life Factors Associated With the Timing of Adiposity Peak and Rebound: A Large Population-Based Longitudinal Study

**DOI:** 10.3389/fped.2021.742551

**Published:** 2021-12-22

**Authors:** Dan Lin, Didi Chen, Jun Huang, Yun Li, Xiaosa Wen, Ling Wang, Huijing Shi

**Affiliations:** ^1^Department of Maternal, Child and Adolescent Health, School of Public Health, Fudan University, Shanghai, China; ^2^Department of School Health, Minhang District Center of Disease Control and Prevention, Shanghai, China; ^3^Minhang Branch, School of Public Health, Fudan University, Shanghai, China; ^4^Department of Child Care, Minhang Maternal and Child Health Center, Shanghai, China; ^5^Shanghai Medical College of Fudan University, Shanghai, China

**Keywords:** adiposity rebound, adiposity peak, body mass index, obesity, growth trajectory

## Abstract

**Background:** The late occurrence of adiposity peak (AP) and the early occurrence of adiposity rebound (AR) are considered the earliest indicators for obesity and its related health conditions later in life. However, there is still limited information for their upstream factors. Therefore, in this study, we aimed to identify the parental and child factors associated with the timing of AP and AR in the early stage of life.

**Methods:** This is a population-based longitudinal study conducted in Shanghai, China. The BMI data of children born between September 2010 and October 2013 were followed from birth to 80 months. Subject-specific body mass index trajectories were fitted by non-linear mixed-effect models with natural cubic spline functions, and the individual's age at AP and AR was estimated. The generalized linear regression models were applied to identify the upstream factors of late occurrence of AP and early occurrence AR.

**Results:** For 7,292 children with estimated AP, boys were less likely to have a late AP [adjusted risk ratio (RR) = 0.83, 95% confidence interval (CI): 0.77–0.90, *p* < 0.001], but preterm born children had a higher risk of a late AP (adjusted RR = 1.25, 95% CI: 1.07–1.47, *p* < 0.01). For 10,985 children with estimated AR, children with breastfeeding longer than 4 months were less likely to have an early AR (adjusted RR = 0.80, 95% CI: 0.73–0.87, *p* < 0.001), but children who were born to advanced-age mothers and who were born small for gestational age had a higher risk of having an early AR (adjusted RR = 1.21, 95% CI: 1.07–1.36, *p* < 0.01; adjusted RR = 1.20, 95% CI: 1.04–1.39, *p* = 0.01).

**Conclusions:** Modifiable pre-birth or early-life factors associated with the timing of AP or AR were found. Our findings may help develop prevention and intervention strategies at the earliest stage of life to control later obesity and the health conditions and diseases linked to it.

## Introduction

Over the past half-century, the prevalence of overweight or obesity in China has rapidly increased, alongside with fast economic growth, globalization, and urbanization (1). The national data suggest that more than half of Chinese adults are now living with overweight or obesity (2). People affected by obesity are at increased risk of various adverse health conditions, many of which are potentially life-threatening (1). Obesity is now a remarkable public health issue in China (2), but the research about upstream determinants or risk factors at the individual level is scarce in the country (1, 2). It is urgent to identify the starting point or predictor early in life for obesity and provide scientific evidence for developing and implementing prevention and management of obesity in China.

Body mass index (BMI) is an indicator of the amount of body fat (3). It does not directly assess body fat, but it is strongly correlated with the gold standard methods for measuring it (3). As a screening tool for overweight and obesity (4), a higher BMI can be an indicator of a greater risk of health problems due to the weight in an individual. The BMI value rapidly increases during the first year of life and then decreases and reaches a nadir between 4 and 6 years of age. After that, it increases again gradually through adolescence and most of adulthood. The highest point of the BMI trajectory in infancy is termed “adiposity peak (AP),” and the second rise during childhood is referred to as “adiposity rebound (AR)” (5). There is growing evidence that the age at AP or AR has a predictive significance for obesity in later life. The current literature has shown that a later AP or an earlier AR is associated with the occurrence of overweight or obesity (6–9) and the associated adverse health outcomes (insulin resistance/type 2 diabetes mellitus and cardiovascular disorders) in childhood, adolescence, or adulthood (6, 7, 9–12).

As vital and early predictors of life-course health risk, experts conducted extensive, in-depth studies on the determinants for the timing of AP and AR during pre-birth and early-life and agreed that there are modifiable and non-modifiable factors associated with them. Researchers have concurred with each other in the view that genetic susceptibility, sex, ethnicity, and birth order were all non-modifiable factors associated with the timing of AP or AR (5, 13–20). However, studies about modifiable factors caused by *in utero* and early-life exposures reported conflicting results regarding factors such as breastfeeding (13, 21, 22) and maternal complication of gestational diabetes mellitus (GDM) (14, 23), leaving them the subject of controversy. Meanwhile, evidence remains scant regarding modifiable factors associated with the timing of AP or AR, which has been reported other than parental obesity or its related conditions (14, 15, 17, 18), pre-eclampsia (14), isolated hyperglycemia (14), birth weight (5, 13), and breastfeeding (13, 14, 19, 20).

We emphasize the role of early-life risk factors in obesity development and the need to provide recommendations to prevent and manage obesity in China. Hence, we generate the hypothesis that some modifiable antenatal or postnatal factors may associate with the timing of AP or AR, which were confirmed to relate to the risks of adverse health conditions later in life. By estimating the timing of AP and AR at the individual level, we aimed to evaluate their association with prenatal, perinatal, and early-life factors in a contemporary, large, population-based longitudinal study.

## Materials and Methods

### Study Population and Data Sources

**Figure 1:**
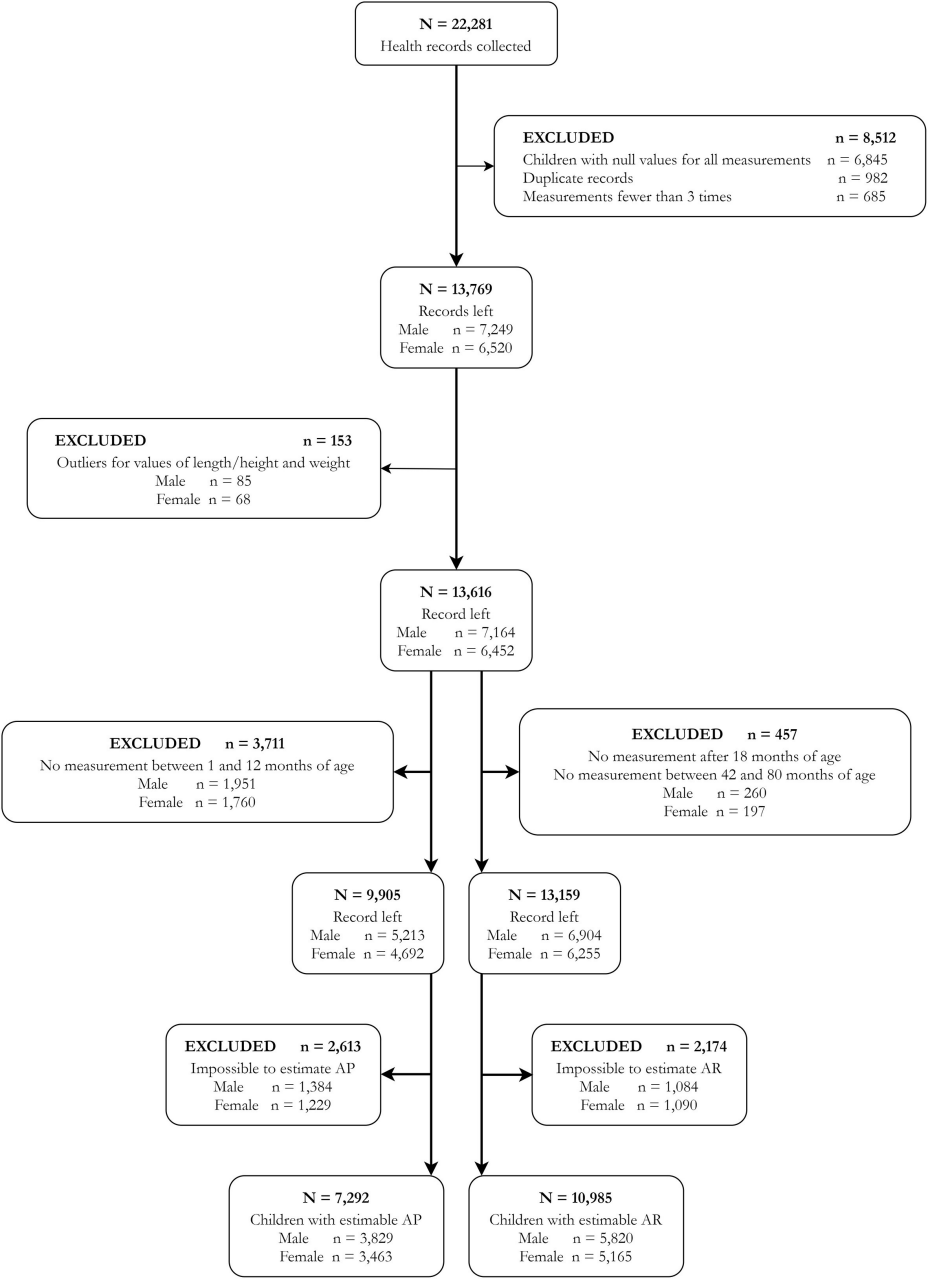
Flow diagram for subject selection.

We used the data from a population-based longitudinal study conducted in the Minhang District of Shanghai, a typical urban area in one of the metropolises of China. Health records of children born between September 2010 and October 2013 in this area were collected from the authorized database (*N* = 22,281). We retrieved data on the age, sex, weight, and height of these children seen for well-child visits or other consultations reported by clinics and hospitals in this area, and retrieved the information concerning the delivery and newborn of the children from birth records. We excluded 8,512 records that met one of the following exclusion criteria: null value for all the measures (*n* = 6,845), duplicate records (*n* = 982), and records with fewer than three measurement times (*n* = 685). We selected only children who had at least three measurement times (weight and length/height at the same measurement, and only one measurement per month) during their 1st and 80th months of age for growth trajectory modeling (24). To avoid influential points, high leverage points, and outliers, we also removed records of children with abnormal growth patterns (*n* = 153). Finally, the number of subjects included in our study was 13,616 (7,164 boys and 6,452 girls) (Figure 1). The Minhang District Center for Disease Control and Prevention Ethics Committee approved this study (EC-2019-011). As the anonymous retrospective data were collected routinely during clinical practice, the requirement for informed consent was waived.

### Timing of AP and AR

Height and weight were measured at each visit at local hospitals or clinics in the Minhang District during infancy and childhood (until the 80th month of age). Bodyweight and length/height were measured by calibrated mechanical weight scales and stadiometers, respectively, and these instruments all passed the inspection by the Shanghai Compulsory Verification Center for Measuring Instruments. Weight was assessed to the nearest 1 g, and the height/body length was measured to the nearest 1 cm. All practices strictly followed the Work Specifications of Children Healthcare, and trained nurses who participated in in-service training performed all measurements following standardized techniques to ensure validity. BMI was calculated as weight in kilograms divided by length or height in meters squared.

All data of multiple observations obtained in early life regarding children's age, weight, and length/height were used to estimate BMI and its trajectories. To guarantee the quality of BMI trajectory fitting, children with no measurement between the 1st and 12th months of age were excluded for AP estimation (*n* = 3,711), and children with no measurement after 18 months of age or between 42 and 84 months of age were excluded for AR estimation (*n* = 457) (Figure 1) (24). The individual-specific BMI trajectory from the 1st to 80th months of age was fitted using non-linear mixed-effect models with natural cubic spline functions for age to capture the non-linear trend (14, 24). Estimates of age at AP and AR were drawn from the BMI-for-age curve for each child. The AP and AR could not be estimated in 2,613 and 2,174 children, respectively, and their ages at AP or AR could not be determined. Based on the final results, 7,292 and 10,985 children have estimated ages at AP and AR, respectively (Figure 1). In this study, the timings of AP and AR were set as binary variables, and the children were categorized into groups of late or non-late AP and early or non-early AR. According to the literature, the age at AP later than 12 months or the age at AR earlier than 48 months has been linked to numerous obesity-related health risks in later life (14, 25–28). Hence, a child who has an AP occurrence later than 12 months of age was defined as being with a late AP, and a child who has an AR occurrence earlier than 48 months of age was defined as being with an early AR in this study.

### The Pre-Birth and Early-Life Factors

Information concerning the delivery and newborn of the children was retrieved from birth records, including sex, gestational age, mode of delivery, and birth weight. Gestational age was measured in weeks, from the first day of the mother's last menstrual cycle to the day of childbirth. Preterm is defined as a child born alive before 37 weeks of gestational age. The delivery modes included vaginal delivery and cesarean section. Children born weighing <2,500 g were defined as low birth weight, and those weighing > 4,000 g were defined as macrosomia. Small for gestational age (SGA) was defined as a birth weight of less than 10th percentile for gestational age, and large for gestational age (LGA) was defined as a birth weight of more than 90th percentile for gestational age (29, 30). Parental information was collected from the hospital birth records, including maternal age, parental education levels, common pregnancy complications, history of childbirth, and multiple gestations. The parental educational level was categorized as university-educated or not (14), and the common complications included gestational anemia, gestational hypertensive disorders, and GDM. Information on breastfeeding and sleep duration of the children was collected from self-administered standardized questionnaires completed by their parents during postpartum follow-ups or collected from pediatric clinical consultation records. The duration of breastfeeding was divided into five groups according to the length of time (longer than 4, 6, 9, or 12 months). The reference group was defined as no breastfeeding initiation or breastfeeding for shorter than 4 months (≤ 4 months). We adopted the World Health Organization (WHO) guideline to reference the sleep duration category during infancy and early childhood (31).

### Statistical Analyses

Characteristics of children are presented as the medians (interquartile range, IQR) for continuous variables and as percentages for categorical variables. Differences in characteristics of children with AP or AR timings were detected by the Wilcoxon rank-sum tests for continuous variables and chi-square tests for categorical variables. Because the detection rate of late AP or early AR occurrence was >15%, we applied the generalized linear model (GLM) to identify the upstream factors, and risk ratios (RRs) with 95% confidence intervals (CIs) were estimated. Univariable regression analyses were first applied to screen the pre-birth and early-life factors. A *p*-value < 0.25 was used as the entry criterion of multivariable regression analyses due to the fact that it is supported in earlier literature and allows for clinically relevant factors to be included (14, 32, 33). After that, the multivariable regression analyses were conducted with factors identified by univariable analyses in the forward stepwise selection approaches. Meanwhile, we also conducted the multivariable analyses by backward stepwise selection approach and simultaneously included all factors identified from the univariable analyses into a regression model (14) as a sensitivity analysis to determine the robustness of the results. The analyses of the upstream factors were based on the children with estimated ages at AP or AR, without imputation for missing data. All tests of hypotheses were 2-sided and conducted at a 0.05 level of significance (*p*-values). All models' fitting was implemented using R version 3.6.2 (34, 35). Statistical analyses were performed in Stata SE 15 (StataCorp LP, Texas, USA).

## Results

### Characteristics of the Study Population

A total of 7,292 children with estimated AP (93,010 times of BMI measurements) and 10,985 children with estimated AR (118,238 times of BMI measurements) were included in this study (Figure 1). Table 1 showed their characteristics by the timings of AP or AR occurrence. Results indicated significant differences in the factors of sex, preterm birth, low birth weight, SGA, multiple gestations (twin), and sleep duration longer than 17 h/day during birth and 3 months of age for children with estimated AP. At the same time, significant differences were found in the factors of advanced maternal age, GDM, breastfeeding longer than 4 months, sleep duration longer than 17 h/day during birth and 3 months of age, and sleep duration longer than 16 h/day during 4 and 11 months of age for children with estimated AR.

**Table 1:** Characteristics of children included in this study.

**Characteristics**	**Children with a late AP**				**Children with an early AR**			
	**Yes**	**No**	**Total**	***p-*value**	**Yes**	**No**	**Total**	***p-*value**
	**(*N* = 3,653)**	**(*N* = 3,639)**	**(*N* = 7,292)**		**(*N* = 4,525)**	**(*N* = 6,460)**	**(*N* = 10,985)**	
Times of valid measurement	14 (12–15)	13 (11–14)	14 (11–14)	<0.001	13 (9–14)	12 (4–14)	12 (6–14)	<0.001
Male—no. (%)	1,758 (48.12)	2,071 (56.91)	3,829 (52.51)	<0.001	2,422 (53.52)	3,398 (52.60)	5,820 (52.98)	0.34
**Parental factor**								
Advanced maternal age (≥35 years)—no. (%)	307 (8.41)	328 (9.04)	635 (8.72)	0.34	294 (6.51)	334 (5.19)	628 (5.73)	<0.01
Mother university-educated—no. (%)	2,358 (70.28)	2,385 (72.34)	4,743 (71.30)	0.06	2,894 (70.16)	4,122 (70.47)	7,016 (70.34)	0.73
Father university-educated—no. (%)	2,420 (71.85)	2,432 (73.63)	4,852 (72.73)	0.10	2,982 (71.91)	4,206 (71.71)	7,188 (71.79)	0.83
Gestational anemia—no. (%)	6 (0.21)	12 (0.43)	18 (0.32)	0.15	8 (0.25)	12 (0.26)	20 (0.26)	0.91
Gestational hypertensive disorders—no. (%)	11 (0.39)	10 (0.36)	21 (0.37)	0.85	9 (0.28)	11 (0.24)	20 (0.26)	0.73
Gestational diabetes mellitus—no. (%)	11 (0.39)	18 (0.64)	29 (0.51)	0.18	26 (0.81)	16 (0.35)	42 (0.54)	<0.01
Multiparous—no. (%)	777 (21.29)	715 (19.68)	1,492 (20.49)	0.09	864 (19.12)	1,210 (18.76)	2,074 (18.91)	0.64
**Child factor**								
Preterm birth—no. (%)	198 (5.43)	118 (3.25)	316 (4.34)	<0.001	165 (3.65)	232 (3.60)	397 (3.62)	0.88
Cesarean delivery—no. (%)	1,695 (46.40)	1,767 (48.61)	3,462 (47.50)	0.06	2,020 (44.64)	2,861 (44.32)	4,881 (44.45)	0.74
Low birth weight—no. (%)	120 (3.28)	71 (1.95)	191 (2.62)	<0.001	119 (2.80)	159 (2.61)	278 (2.69)	0.54
Macrosomia—no. (%)	197 (5.39)	233 (6.40)	430 (5.90)	0.07	282 (6.40)	364 (5.78)	646 (6.03)	0.18
Small for gestational age—no. (%)	216 (6.49)	159 (4.87)	375 (5.69)	0.01	276 (6.74)	359 (6.12)	635 (6.38)	0.21
Large for gestational age—no. (%)	323 (9.41)	373 (10.73)	696 (10.07)	0.07	426 (10.04)	584 (9.59)	1,010 (9.77)	0.45
Twin—no. (%)	77 (2.82)	40 (1.45)	117 (2.13)	<0.001	60 (1.67)	94 (1.77)	154 (1.73)	0.72
Breastfeeding duration—no. (%)								
>4 months	724 (28.35)	712 (26.22)	1,436 (27.25)	0.08	768 (27.03)	1,018 (32.59)	1,786 (29.94)	<0.001
>6 months	285 (11.16)	261 (9.61)	546 (10.36)	0.07	321 (11.30)	403 (12.90)	724 (12.14)	0.06
>9 months	NA				91 (3.20)	97 (3.10)	188 (3.15)	0.83
>12 months					47 (1.65)	44 (1.41)	91 (1.53)	0.44
Sleep duration—no. (%)								
<14 h/day during 0–3 months of age	42 (1.20)	56 (1.56)	98 (1.38)	0.20	47 (1.25)	66 (1.50)	113 (1.39)	0.33
>17 h/day during 0–3 months of age	1,426 (40.86)	1,329 (36.98)	2,755 (38.89)	<0.001	1,309 (34.75)	1,640 (37.35)	2,949 (36.15)	0.02
<12 h/day during 4–11 months of age	NA				8 (0.20)	18 (0.40)	26 (0.31)	0.10
>16 h/day during 4–11 months of age					52 (1.33)	85 (1.91)	137 (1.64)	0.04
<11 h/day during 12–24 months of age					20 (0.50)	22 (0.50)	42 (0.50)	0.99
>14 h/day during 12–24 months of age					129 (3.24)	153 (3.47)	282 (3.36)	0.55

*AP, adiposity peak; AR, adiposity rebound; NA, not applicable*.

### Factors Associated With the Late Occurrence of AP or Early Occurrence of AR

The results of univariate regression analyses are shown in Table 2. The upstream factors related to a late AP occurrence and selected into the multivariable model were parental education levels, gestational anemia, GDM, multiparous, sex, preterm birth, cesarean delivery, multiple gestations, low birth weight, macrosomia, SGA, breastfeeding duration longer than 4 and 6 months, sleep duration shorter than 14 h/day during birth and 3 months of age, and longer than 17 h/day during birth and 3 months of age. The forward stepwise regression was run, and the multivariable regression analysis showed that sex and preterm birth were factors associated with the late occurrence of AP. Boys were less likely to have a late AP than girls (adjusted RR = 0.83, 95% CI: 0.77–0.90, *p* < 0.001), and children who were born preterm was associated with a higher risk of late AP occurrence (adjusted RR = 1.25, 95% CI: 1.07–1.47, *p* < 0.01). Sensitivity analyses were performed, and the results were similar to those in the forward stepwise regression (the results of backward stepwise regression and those for the regression model simultaneously included all factors identified from the univariable analyses are presented in Supplementary Tables S1, S2).

**Table 2:** Univariate regression analyses of factors associated with the late occurrence of AP or early occurrence of AR.

**Characteristics**	**Late AP**			**Early AR**		
	**Crude RR**	**95% CI**	***p-*value**	**Crude RR**	**95% CI**	***p-*value**
**Parental factor**						
Advanced maternal age (≥35 years)	0.96	0.88–1.04	0.35	1.15	1.05–1.25	<0.01
Mother university-educated	0.95	0.90–1.00	0.06	0.99	0.94–1.04	0.73
Father university-educated	0.96	0.91–1.01	0.10	1.01	0.95–1.06	0.83
Gestational anemia	0.66	0.34–1.27	0.22	0.97	0.57–1.66	0.91
Gestational hypertensive disorders	1.04	0.69–1.57	0.85	1.09	0.67–1.77	0.73
Gestational diabetes mellitus	0.75	0.47–1.20	0.23	1.50	1.18–1.91	<0.01
Multiparous	1.05	0.99–1.11	0.09	1.01	0.96–1.07	0.64
**Child factor**						
Male	0.84	0.80–0.88	<0.001	1.02	0.98–1.07	0.34
Preterm birth, <37 weeks of gestational age at delivery	1.27	1.16–1.38	<0.001	1.01	0.90–1.14	0.88
Cesarean delivery	0.96	0.91–1.00	0.06	1.01	0.96–1.05	0.74
Twin	1.33	1.17–1.52	<0.001	0.96	0.79–1.18	0.73
Low birth weight	1.26	1.13–1.41	<0.001	1.04	0.91–1.20	0.54
Macrosomia	0.91	0.82–1.01	0.08	1.06	0.97–1.17	0.17
Small for gestational age	1.15	1.05–1.26	<0.01	1.06	0.97–1.16	0.21
Large for gestational age	0.95	0.78–1.14	0.56	1.03	0.95–1.11	0.45
Breastfeeding duration						
>4 months	1.06	0.99–1.12	0.08	0.87	0.82–0.92	<0.001
>6 months	1.09	1.00–1.18	0.06	0.92	0.85–1.01	0.07
>9 months	NA			1.02	0.88–1.18	0.83
>12 months				1.09	0.89–1.33	0.42
Sleep duration						
<14 h/day during 0–3 months of age	0.87	0.69–1.09	0.23	0.90	0.72–1.12	0.35
>17 h/day during 0–3 months of age	1.09	1.04–1.14	<0.01	0.94	0.90–0.99	0.02
<12 h/day during 4–11 months of age	NA			0.66	0.37–1.17	0.15
>16 h/day during 4–11 months of age				0.81	0.65–1.00	0.05
<11 h/day during 12–24 months of age				1.00	0.73–1.38	0.99
>14 h/day during 12–24 months of age				0.96	0.85–1.09	0.56

*AP, adiposity peak; AR, adiposity rebound; RR, risk ratio; CI, confidence interval; NA, not applicable*.

The upstream factors related to an early AR occurrence and selected into the multivariable model were advanced maternal age (≥35 years), GDM, SGA, breastfeeding duration longer than 4 and 6 months, and sleep duration longer than 17 h/day during birth and 3 months of age, sleep duration shorter than 12 h/day during 4 and 11 months of age, and sleep duration longer than 16 h/day during 4 and 11 months of age. The forward stepwise regression was run, and the multivariable analysis showed that breastfeeding duration longer than 4 months, advanced maternal age, and SGA were the factors associated with the timing of AR. Children with breastfeeding longer than 4 months were less likely to have an early AR than those who had no breastfeeding or had breastfeeding for shorter than 4 months (adjusted RR = 0.80, 95% CI: 0.73–0.87, *p* < 0.001). Children who were born to advanced-age mothers and who were born SGA had a higher risk of having an early AR (adjusted RR = 1.21, 95% CI: 1.07–1.36, *p* < 0.01; adjusted RR = 1.20, 95% CI: 1.04–1.39, *p* = 0.01). Sensitivity analyses were also performed, and results were similar to those in the forward stepwise regression (Supplementary Tables S1, S2).

## Discussion

In this large, population-based longitudinal study, we found the parental and early-life factors for the occurrence of late AP and early AR in children who were born after the first decade in this century and grew up in a metropolis of China. The upstream factor, such as sex, preterm birth, breastfeeding duration, advanced maternal age, or SGA, was associated with the timing of AP or AR.

In line with the previous studies, we found that females were more likely to have a late AP than males (5, 13, 14). As a non-modifiable factor, the sex difference in physical development during infancy is difficult to be explained by hormones, nutrition status, or physical activity levels. Hence, it may be attributed to a genetic difference. Although a late AP was suggested to be associated with later adiposity, no evidence supported that females are at a higher risk of being overweight or obese at any stage of their life.

Children born preterm were found to have a higher risk of late AP occurrence in our study. The growth pattern of preterm infants is usually catch-up growth. Catch-up growth refers to the rapid growth of preterm infants that minimizes the size difference with term counterparts. It is usually noted in the weight and length and occurs during the first 2–3 years of life (36, 37). In this study, a late AP means the peak of a BMI curve occurred after 12 months of age. We can speculate that the preterm born children still had a delay in reaching the peak BMI value within infancy, and the longer time left to BMI increase contributes to the larger magnitude of BMI value, which tracks to the later life. Experiments on animal models showed that mice with catch-up growth would be susceptible to the adverse effects of an obesity-inducing diet and favor the development of obesity in adulthood (38, 39). For humans, children with catch-up growth were fatter and had more central fat distribution at 5 years of age, and had the risk of visceral obesity, insulin resistance, and glucose intolerance in adulthood (40, 41). Therefore, future studies are encouraged to explore the mediating effect of catch-up growth between preterm birth and AP timing. Certainly, preterm birth also appeared to be independently associated with an increased risk of developing cardiovascular disease in adolescence and type 2 diabetes in adulthood (42, 43).

In the crude model for factors associated with the late occurrence of AP, the identified factors also included multiple gestations (twin), low birth weight, and SGA (all *p*-value < 0.01). Although the associations could not be observed in the adjusted model, the factors all pointed to the catch-up growth pattern. The result indicated that growth monitoring should be implemented for children with intrauterine growth restriction to identify catch-up growth, and the intervention of obesity should be provided early in childhood. Previous studies also reported that isolated gestational hyperglycemia, a larger birth weight, and breastfeeding non-initiation were associated with a delayed AP (5, 13, 14, 19, 20, 44, 45). However, these associations could not be observed in this study.

Results also demonstrated that children with breastfeeding longer than 4 months had later AR than their counterparts who had no breastfeeding or had breastfeeding for shorter than 4 months. However, this association could not be found among children with breastfeeding longer than 6, 9, or 12 months. It is worth noting that two studies from Europe did not report the association between early AR occurrence and breastfeeding duration longer than 6 months, too. These two studies suggested that children of European ancestry receiving 3–5 months of exclusive breastfeeding had later AR only in females, but no observation was identified in males (19, 20). Furthermore, although the association between breastfeeding and AR timing is a topic of general interest, whether breastfeeding protects children from early AR remains determined (46–48). As there are increasing challenges (49) of the widely accepted opinion that a longer duration of breastfeeding is associated with slower growth and less weight gain than formula feeding in young children (50), it is a necessity for future studies to examine the effect of breastfeeding in terms of its duration.

No previous study reported the association between advanced maternal age and early AR. The immediate neonatal outcomes of advanced maternal age included pre-eclampsia, intrauterine growth restriction, preterm birth, and stillbirth (51–54), and the long-term outcomes were cardiovascular disease, obesity, and certain types of cancers in adulthood (55–58). A study from Kuwait suggested that childhood obesity was associated with advanced maternal age at pregnancy (59). However, a recent study from China has shown that maternal age younger than 28 years could predict pediatric overweight or obesity (60). Other studies also supported that although children of mothers with an advanced maternal age exhibited a higher BMI, mother's age at pregnancy has not been identified as a predictor of overweight for children (61, 62). Based on all known evidence, we infer that the association between advanced maternal age and the early occurrence of AR was mediated by immediate neonatal outcomes such as intrauterine growth restriction or preterm birth.

Children born SGA are also tied to an early AR in this study. Researches summarized that SGA was associated with early AR, obesity, metabolic syndrome, or non-communicable diseases in later life (63). A Japanese study suggested that about 7% of children with SGA had AR before 3 years of age (64). Compared with children born appropriate for gestational age and born large for gestational age, children born SGA seemed to have the earliest timing of AR (65).

### Strengths and Limitations

The strengths of this study include the large sample size and the standardized and quality-assured data collection processes that were conducted over a long-time span from pregnancy to middle childhood. However, several limitations have impacted our results. First of all, AP and AR timing estimations used for analysis was not from the observation records and might lead to inaccuracy. Also, there was a possibility of recall bias since the data of some variables were self-reported. Meanwhile, due to one of our hypotheses that the birth weight would be an associated factor, we did not include the birth weight/length/BMI when deriving individuals' BMI growth trajectories, even if it is a strong determinant of subsequent growth. Additionally, we did not differentiate breastfeeding by exclusive breastfeeding or not due to the loss of information. The breastfeeding in this study referred to any breastfeeding (includes nonexclusive and exclusive). Furthermore, since the subjects were almost Chinese Han children born and lived in an economically developed area, generalization of these results to other ethnic groups or regions should be performed with caution. Finally, although preterm birth, advanced maternal age, and SGA were risk factors identified in our study, their clinical impact could be limited. Results in Table 1 showed that in our population, only 5.4% of children with a late AP were born preterm, 6.5% of children with an early AR were born to advanced-age mothers, and 6.7% were born SGA. Modification of these factors could just have limited impact on children's timing of AP and AR.

In conclusion, in this population-based longitudinal study, the timing of AP or AR occurrence was found to be explained by factors arising in pregnancy and early infancy. Our study underscores a critical need for interventions to change modifiable factors during the early stage of life to affect the AP or AR occurrence time and may even affect adiposity and its complications in later life. However, the timing of AP and AR is not fully explained by the evaluated factors included in our study. Therefore, further studies are required for the investigation of other upstream factors.

## Data Availability Statement

The datasets presented in this article will be available for investigators after approval by Fudan University and the Minhang District Center for Disease Control and Prevention. Requests to access the datasets should be directed to Huijing Shi, hjshi@fudan.edu.cn.

## Ethics Statement

The studies involving human participants were reviewed and approved by the Minhang District Center for Disease Control and Prevention Ethics Committee. Written informed consent from the participants' legal guardian/next of kin was not required to participate in this study in accordance with the national legislation and the institutional requirements.

## Author Contributions

HS, DL, and DC conceptualized and designed the study, contributed to data analysis, drafted the initial manuscript, and reviewed and revised the manuscript. LW and XW helped with study design, overviewed the study, coordinated data acquisition, and contributed to data analysis. JH and YL coordinated and supervised data collection, served as data managers at their institutions, and critically reviewed the manuscript. All authors critically reviewed and approved the final manuscript.

## Funding

This study was supported by the Special Foundation of Basic Science and Technology Resources Survey from the Ministry of Science and Technology of China (2019FY101004), the Fifth Round of the Three-Year Public Health Action Plan of Shanghai from the Shanghai Municipal Health Commission (GWV-10.1-XK08), and the Fudan-Minhang Health Consortium Cooperation Project from the Minhang Branch, School of Public Health, Fudan University, Shanghai (2019FM11).

## Conflict of Interest

The authors declare that the research was conducted in the absence of any commercial or financial relationships that could be construed as a potential conflict of interest.

## Publisher's Note

All claims expressed in this article are solely those of the authors and do not necessarily represent those of their affiliated organizations, or those of the publisher, the editors and the reviewers. Any product that may be evaluated in this article, or claim that may be made by its manufacturer, is not guaranteed or endorsed by the publisher.
