# Supplementary material for: Pre-Birth and Early-Life Factors Associated With the Timing of Adiposity Peak and Rebound: A Large Population-Based Longitudinal Study
Source: Front Pediatr. 2021 Dec 22;9:742551. doi: 10.3389/fped.2021.742551 (PMC8727998; doi:10.3389/fped.2021.742551)
Supplement: Supplementary file 1 [file Data_Sheet_1.docx]

**Table S1 Backward stepwise regressions for factors associated with the late occurrence of AP or early occurrence of AR**

| **Characteristics** | **Late AP** | | |  | **Early AR** | | |
| --- | --- | --- | --- | --- | --- | --- | --- |
|  | **Adjusted RR** | **95% CI** | ***P-*value** |  | **Adjusted RR** | **95% CI** | ***P-*value** |
| **Parental factor** |  |  |  |  |  |  |  |
| Advanced maternal age (>= 35 years) | NA | | |  | 1.21 | 1.07-1.36 | <0.01 |
| **Child factor** |  |  |  |  |  |  |  |
| Male | 0.83 | 0.77-0.90 | <0.001 |  | NA | | |
| Preterm birth, <37 weeks of gestational age at delivery | 1.25 | 1.03-1.52 | <0.01 |  |  |  |  |
| Small for gestational age | NA | | |  | 1.20 | 1.04-1.39 | 0.01 |
| Breastfeeding duration > 4 months |  |  |  |  | 0.80 | 0.73-0.87 | <0.001 |

*AP, adiposity peak; AR, adiposity rebound; RR, risk ratio; CI, confidence interval; NA, not applicable.*

*The* *backward stepwise regressions were conducted.*

**Table S2** **Multivariate regression analyses of factors associated with the late occurrence of AP or early occurrence of AR**

| **Characteristics** | **Late AP** | | |  | **Early AR** | | |
| --- | --- | --- | --- | --- | --- | --- | --- |
|  | **Adjusted RR** | **95% CI** | ***P-*value** |  | **Adjusted RR** | **95% CI** | ***P-*value** |
| **Parental factor** |  |  |  |  |  |  |  |
| Advanced maternal age (>= 35 years) | NA | | |  | 1.20 | 1.07-1.35 | <0.01 |
| Mother university-educated | 0.97 | 0.84-1.12 | 0.65 |  | NA | | |
| Father university-educated | 1.00 | 0.87-1.15 | 0.99 |  |  |  |  |
| Gestational anemia | 0.59 | 0.20-1.80 | 0.36 |  |  |  |  |
| Gestational diabetes mellitus | 1.09 | 0.64-1.85 | 0.75 |  | 1.14 | 0.74-1.75 | 0.54 |
| Multiparous | 0.99 | 0.89-1.11 | 0.89 |  | NA | | |
| **Child factor** |  |  |  |  |  |  |  |
| Male | 0.83 | 0.77-0.90 | <0.001 |  | NA | | |
| Preterm birth, <37 weeks of gestational age at delivery | 1.25 | 1.03-1.52 | <0.01 |  |  |  |  |
| Cesarean delivery | 1.01 | 0.93-1.09 | 0.84 |  |  |  |  |
| Twin | 1.19 | 0.94-1.50 | 0.15 |  |  |  |  |
| Low birth weight | 0.97 | 0.75-1.27 | 0.84 |  |  |  |  |
| Macrosomia | 0.83 | 0.41-1.69 | 0.61 |  | 0.74 | 0.44-1.25 | 0.27 |
| Small for gestational age | 1.11 | 0.94-1.32 | 0.20 |  | 1.20 | 1.04-1.38 | 0.01 |
| Breastfeeding duration |  |  |  |  |  |  |  |
| > 4 months | 1.02 | 0.93-1.13 | 0.68 |  | 0.79 | 0.71-0.88 | <0.001 |
| > 6 months | 1.02 | 0.88-1.18 | 0.76 |  | 1.02 | 0.88-1.20 | 0.72 |
| Sleep duration |  |  |  |  |  |  |  |
| < 14 hours per day during 0-3 months of age | 0.71 | 0.46-1.12 | 0.14 |  | NA | | |
| > 17 hours per day during 0-3 months of age | 1.03 | 0.95-1.12 | 0.47 |  | 0.94 | 0.87-1.01 | 0.08 |
| < 12 hours per day during 4-11 months of age | NA | | |  | 0.87 | 0.48-1.60 | 0.66 |
| > 16 hours per day during 4-11 months of age |  |  |  |  | 0.98 | 0.66-1.46 | 0.92 |

*AP, adiposity peak; AR, adiposity rebound; RR, risk ratio; CI, confidence interval; NA, not applicable.*

*All factors identified from the univariable analyses were simultaneously included in the multivariable analyses.*
